# A cardiac and subcutaneous canine dirofilariosis outbreak in a kennel in central France

**DOI:** 10.1051/parasite/2019073

**Published:** 2019-12-16

**Authors:** Younes Laidoudi, David Ringot, Stéphanie Watier-Grillot, Bernard Davoust, Oleg Mediannikov

**Affiliations:** 1 Aix Marseille Univ, IRD, APHM, MEPHI, IHU Méditerranée Infection 13385 Marseille France; 2 IHU Méditerranée Infection 13385 Marseille France; 3 French Military Health Service, 33e Veterinary Group 37076 Tours France; 4 Animal Epidemiology Working Group of the Military Health Service 13014 Marseille France; 5 French Armed Forces Centre for Epidemiology and Public Health (CESPA) 13014 Marseille France

**Keywords:** *Dirofilaria immitis*, *Dirofilaria repens*, Heartworm disease, PCR, Military working dog, France

## Abstract

Canine dirofilarioses are nematode infections caused by two species of the genus *Dirofilaria: D. immitis* and *D. repens*. We describe here an outbreak of *D. immitis* and *D. repens* infection in military working dogs (MWDs) housed in a kennel in the Indre department (centre of France). Out of a total of 17 dogs, 6 (35.2%) tested positive for *D. immitis*, *D. repens* or both parasites. Infested dogs were treated and prophylactic measures were implemented for the entire kennel staff. To our knowledge, this is the first documented description of an outbreak of canine cardiopulmonary dirofilariasis in the center of France, unlike in the south of this country, where *D. immitis* and *D. repens* dirofilariasis are enzootic. In France, as mosquito vectors expand their territory and new non-native vectors are introduced, it is likely that the distribution area of these two diseases of domestic and wild carnivores will be wider and underestimated.

## Abstract (fr)

Les dirofilarioses canines sont des infections à nématodes causée par deux espèces du genre *Dirofilaria*, *D. immitis* et *D. repens*. Nous décrivons ici un foyer d’infection à *D. immitis* et *D. repens* chez des chiens militaires hébergés dans un chenil dans le département de l’Indre (centre de la France). Sur un total de 17 chiens, 6 (35,2 %) ont été testés positifs pour *D. immitis*, pour *D. repens* ou pour les deux parasites. Les chiens infestés ont été traités et des mesures prophylactiques ont été mises en place pour tout le personnel du chenil. À notre connaissance, il s’agit de la première description documentée d’un foyer de dirofilariose cardiopulmonaire canine dans le centre de la France, contrairement au sud du pays, où les dirofilarioses à *D. immitis* et *D. repens* sont enzootiques. En France, à mesure que les moustiques vecteurs élargissent leur territoire et que de nouveaux vecteurs non indigènes sont introduits, il est probable que la zone de distribution de ces deux maladies des carnivores domestiques et sauvages sera plus étendue et sous-estimée.

## Introduction

*Dirofilaria immitis* (Leidy, 1856) [23] and *Dirofilaria repens* Railliet & Henry, 1911 [33] are mosquito-borne filarioids (Nematoda: Onchocercidae) infecting wild and domestic mammals of different orders with canids as the predominant definitive hosts. Adult worms of *D. immitis* with a smooth cuticle (measuring between 12 cm and 30 cm in length) colonize the pulmonary arteries and right heart cavities, whereas adult worms of *D. repens,* with a finely striated cuticle, (measuring between 5 cm and 17 cm in length) are located in the subcutaneous tissues [11, 26, 28]. About 120 days after infection of the mammalian host, the viviparous female can be fertilized and produces mobile embryos called microfilariae. Microfilariae (L1 stage) parasitize the blood until being ingested by the mosquito vector (Culicidae) during a blood meal taken on a microfilaremic host. Inside the vector, microfilariae first develop into larval stage 1 (L1), then molt into larval stage 2 (L2), and finally molt into third-stage larvae (L3), which is the infective stage. Clinically, *D. immitis* infection (canine heartworm disease) can remain unapparent for a long time. Symptoms are mainly dominated by right heart failure, ranging from exercise intolerance and fatigue to cardiac decompensation with swelling and possible acute pulmonary oedema [12, 26, 29]. In contrast, the infestation by *D. repens* often passes unnoticed. Dogs infected with *D. repens* microfilariae may sometimes develop subcutaneous nodules where adult worms encyst. Sometimes, the disease manifests with more symptoms, such as pruritus and skin changes [38].

Canine dirofilariosis has been known in France for a long time. It was in 1679 that Panhot highlighted heartworm in a dog native to the Dombes region (near Lyon) [30]. Over the last century, several studies have shown the importance of this parasitosis in the Mediterranean region [8, 18]. At the beginning of the 1980s, the infestation rate of military working dogs (MWDs) was high, particularly in Corsica and the Bouches-du-Rhône department [5, 8]. Out of 180 MWDs tested in 1988, 67 (37%) carried microfilariae in the blood, including 17 cases of *D. immitis*, 2 of *D. repens*, 15 mixed infestations and 3 doubtful ones [5]. A screening test carried out on 85 MWDs from northern France (Brittany and Normandy) was negative [6]. The MWDs with heartworm disease may lose their operational fitness, causing an operational impact for the Armed Forces. In fact, MWDs military dogs are used for the detection of explosive devices as well as to secure military sites. The eradication of canine heartworm disease among military dogs in south-eastern France was made possible by the implementation, for the first time in France, of chemoprophylaxis based on the use of ivermectin, completed by a conclusive trial on the efficacy of melarsomine in treating adult *Dirofilaria* [4]. The first demonstration of the long-term effectiveness of this protocol was made in 2015 by comparing the incidence rates of *D. immitis* infestation in MWDs from a kennel located in Corsica and in co-located civilian dogs (the civilian kennel was less than 15 km from the military kennel). The estimated prevalence in civilian dogs was 40.4% (19/47), while no cases were identified in MWDs [36]. Currently, canine dirofilarioses chemoprophylaxis is applied to all MWDs deployed on missions outside mainland France, as well as to MWDs from mainland France located in south-eastern France, during the period of vector activity (May–November). In the present study, we revealed the existence of an indigenous outbreak of *D. immitis* and *D. repens* infestation in the French department of Indre. To the best of our knowledge, this is the first documented description of an outbreak of canine cardiopulmonary dirofilariosis in this region of France.

## Materials and methods

### Dogs

In October 2018, we performed blood tests on the 17 apparently healthy dogs in the military kennel located near the city of Rosnay (46°42′47″N, 1°14′39″E), in Indre, central France. The commune is located in the “Parc naturel régional de la Brenne”. The MWDs, including 11 Belgian Malinois and 6 German Shepherds, were all males aged 2–10 years, with a median of 5 years. The duration of the dogs’ presence in the kennel ranged from 6 months to 8.5 years, with a median of 4 years. MWDs, including dogs from the Rosnay kennel, are purchased abroad, mainly in eastern European countries (Germany and Poland, in particular). They arrived at the kennel at about 2 years of age. They received regular veterinary care. From May to October, they benefit from a treatment against ectoparasites, but which has no repellent effect on mosquitoes. For the purpose of the study, two blood samples of 4–5 mL volume were taken from each dog and collected from a dry tube and a citrated tube, respectively. Serum was collected after centrifugation (10 min, 3000 g). Each animal sampled was examined clinically.

### Ethics statement

Blood samples were collected in accordance with the requirements of the Animal Ethics Procedures of French veterinarians and with the consent of the owner of the animals (French Armed Forces).

### Direct detection of microfilariae

Modified Knott’s staining was chosen to identify canine blood microfilaria [21, 25]. One millilitre of citrated blood was mixed with 9 mL of hemolyzing solution (2% acetic acid) in a 15 mL tube, followed by centrifugation for minutes at 500 rpm. The supernatant was removed; the sediment was stained with methylene-blue, transferred to a slide, and covered with a cover slip.

### Heartworm antigen detection

Heartworm antigen detection was performed using two rapid diagnostic serological tests marketed in France and targeting the antigen secreted by the adult female worm: (i) a WITNESS^®^ Dirofilaria test (Zoetis, Lyon, France), based on immunomigration (RIM^®^) technology, and (ii) a DiroCHEK^®^ test (Zoetis, Lyon, France), based on an enzyme-linked immunosorbent assay method known as a sandwich ELISA. Both tests provide rapid results, as well as high sensitivity (99% for WITNESS^®^ Dirofilaria and 98% for DiroCHEK^®^) and specificity (94% for WITNESS^®^ Dirofilaria and 96% for DiroCHEK^®^) [17].

### Molecular detection of filaria and the Wolbachia endosymbiont of filaria

DNA was extracted from 100 μL of citrated blood, after 4-hour digestion at 56 °C, using an equal volume of buffer G2 supplemented with 15% proteinase K. The extraction was performed using the Biorobot EZ1 System with the EZ1 DNA tissue kit (Qiagen, Courtaboeuf, France), in line with the manufacturer’s instructions. DNA was eluted in a final volume of 100 μL and stored at −20 °C. All samples were screened for filaria and their *Wolbachia* complex, using the fast typing approach which consists in a pan-filarial 28S-based qPCR system, followed by a triplex COI-based system targeting *D. immitis*, *D. repens* and *Acanthocheilonema reconditum,* and the duplex ftsZ-based system, targeting specifically the *Wolbachia* endosymbiont of *D. immitis* and that of *D. repens* (Table 1) [22]*.* Once the specific filarial DNA has been revealed by the qPCR triplex, the sample is considered positive, and the detection of *Wolbachia* DNA confirms infection by *D. immitis* or *D. repens* related to this strain.

**Table 1:** Primers and probes used in this study.

System name	Primer & probe name	Sequences 5′–3′	Specificity	References
Pan-fil 28S qPCR-based system	qFil-28S-F	TTGTTTGAGATTGCAGCCCA	Filariae	[22]
	qFil-28S-P	6FAM-5′-CAAGTACCGTGAGGGAAAGT-3′-TAMRA		
	qFil-28S-R	GTTTCCATCTCAGCGGTTTC		
All-Wol 16S qPCR-based system	all.Wol.16S.301-F	TGGAACTGAGATACGGTCCAG	Wolbachieae	
	all.Wol.16S.347-P	6FAM-5′-AATATTGGACAATGGGCGAA-3′-TAMRA		
	all.Wol.16S.478-R	GCACGGAGTTAGCCAGGACT		
Triplex TaqMan COI qPCR-based system	Fil.COI.749-F	CATCCTGAGGTTTATGTTATTATTTT		
	D.imm.COI.777-P	6FAM-CGGTGTTTGGGATTGTTAGTG-TAMRA	*Dirofilaria immitis*	
	D.rep.COI.871-P	6VIC-TGCTGTTTTAGGTACTTCTGTTTGAG-TAMRA	*Dirofilaria repens*	
	A.rec.COI.866-P	Cy5-TGAATTGCTGTACTGGGAACT-BHQ-3	*Acanthocheilonema reconditum*	
	Fil.COI.914-R	CWGTATACATATGATGRCCYCA		
Duplex Wol-Diro ftsZ qPCR-based system	WDiro.ftsZ.490-F	AAGCCATTTRGCTTYGAAGGTG	*Wolbachia* endosymbiont of *D. immitis* and *D. repens*	
	WDimm.ftsZ.523-P	6FAM-CGTATTGCAGAGCTCGGATTA-TAMRA		
	WDrep.ftsZ.525-P	6VIC-CATTGCAGAACTGGGACTGG-TAMRA		
	WDiro.ftsZ.600-R	AAACAAGTTTTGRTTTGGAATAACAAT		
Duplex HWs COI qPCR-based system	Hw.COI.723-F	TCAGCATTTGTTTTGGTTTTT		
	D.imm.COI.777-P	6FAM-CGGTGTTTGGGATTGTTAGTG-TAMRA	*D. immitis*	
	A.vas.COI.813-P	6VIC-TGACTGGGAAGAAGGAGGTG-TAMRA	*Angiostrongylus vasorum*	
	Hw.COI.950-R	GCASTAAAATAAGYACGAGWATC		
Pan-Nematoda primers 18S PCR-based system	Fwd.18S.631	TCGTCATTGCTGCGGTTAAA	Nematoda	This study
	Rwd.18S.1825r	GGTTCAAGCCACTGCGATTAA		

### Sequencing analysis and phylogenetic genotyping of filaria

Samples harbouring a single DNA of filaria were subjected to sequencing analysis. The pan-Nematoda primers named Fwd.18S.631 & Rwd.18S.1825r (Table 1) were designed and customized to amplify an 1127–1155-bp fragment from the 18S rRNA gene. PCR reactions were carried out in a total volume of 50 μL, consisting of 25 μL of AmpliTaq Gold master mix, 18 μL of ultra-purified water DNAse-RNAse free, 1 μL of each primer and 5 μL of DNA template. The thermal cycling conditions were: incubation step at 95 °C for 15 min, 40 cycles of 1 min at 95 °C, 30 s at 54 °C for the melting temperature, and one and half minutes for the elongation time at 72 °C, followed by a final extension of 5 min at 72 °C. PCR amplification was performed in a Peltier PTC-200 model thermal cycler (MJ Research Inc., Watertown, MA, USA). The DNA generated through the PCR reaction was purified by filtration using a NucleoFast^®^ 96 PCR DNA purification plate, and was then amplified using a BigDye^®^ Terminator v3.1 Cycle Sequencing Kit (Applied Biosystems, Foster City, CA, USA). The BigDye PCR products were purified on the Sephadex G-50 Superfine gel filtration resin prior to sequencing on the ABI Prism 3130XL. Nucleotide sequences were assembled and corrected using ChromasPro 2.0.0, then aligned against close reference sequences of filarioids species, representative members of Onchocercidae available in GenBank. The alignment was performed using the ClustalW application within BioEdit v.7.2.5 [16]. The maximum likelihood phylogenetic tree was inferred on MEGA6 [37], based on the Kimura 3-substitution-type model [20].

## Results and discussion

Table 2 presents the results of the nine analyses carried out on the blood of 17 MWDs of the Rosnay kennel. For canine dirofilariosis, we observed 35.2% (6/17) positive dogs. The Knott test conducted on dog No. 3 revealed the presence of *D. immitis* microfilariae. DNA and *Wolbachia* complex qPCRs tests as well as the two serological tests gave positive results. Five dogs had mixed infestation with *D. immitis* and *D. repens* (dogs Nos. 2, 7, 8, 10 and 13). *Dirofilaria repens* DNA was identified in dogs Nos. 2, 10 and 13. Furthermore, both *Wolbachia* genotypes known to be associated with *D. immitis* and *D. repens* were also detected in the same samples. Two samples (Nos. 2 and 13) provided positive results with both serological tests. One dog (No. 7) was positive for both *D. immitis* and *D. repens* microfilariae by the Knott test, the PCR test for both *Dirofilaria* and *Wolbachia* DNA, and by ELISA; whereas, it was negative by the immunomigration test. Finally, dog No. 8 was positive for *D. immitis* and *D. repens* and *Wolbachia* DNA; and for both serological tests. All samples were free of *A. reconditum* infection.

**Table 2:** Screening for dirofilariosis in a military kennel in the Indre department (central France).

Dog number (No.)	Breed*	Age (year)	Kennel presence time (year)	Parasitological diagnosis: Knott test	Serological screening		Molecular detection of filarial DNA using the qPCR Pan-Filaria 28S	Genotyping: 18S rRNA gene	Molecular identification of filarial species using a COI Triplex qPCR-based system			Molecular identification of *Wolbachia* genotypes using ftsZ duplex qPCR-based system		Diagnosis
					Witness® Dirofilaria	DiroCHEK®			*Dirofilaria immitis*	*Dirofilaria repens*	*Acanthocheilonema reconditum*	*Wolbachia* endosymbiont of *D. immitis*	*Wolbachia* endosymbiont of *D. repens*	
1	BSM	10	8.5	Neg.	Neg.	Neg.	Neg.	NE**	Neg.	Neg.	Neg.	Neg.	Neg.	Healthy dog
2	BSM	7	6	*D. repens*	Pos.	Pos.	Pos.	*D. repens*	Neg.	Pos.	Neg.	Pos.	Pos.	Occult heartworm and subcutaneous dirofilariosis
3	GS	6	5	*D. immitis*	Pos.	Pos.	Pos.	*D. immitis*	Pos.	Neg.	Neg.	Pos.	Neg.	Heartworm disease
4	BSM	6	5	Neg.	Neg.	Neg.	Neg.	NE	Neg.	Neg.	Neg.	Neg.	Neg.	Healthy dog
5	BSM	6	5	Neg.	Neg.	Neg.	Neg.	NE	Neg.	Neg.	Neg.	Neg.	Neg.	Healthy dog
6	BSM	6.5	5	Neg.	Neg.	Neg.	Neg.	NE	Neg.	Neg.	Neg.	Neg.	Neg.	Healthy dog
7	GS	6	4	*D. immitis* + *D. repens*	Neg.	Pos.	Pos.	NE	Pos.	Pos.	Neg.	Pos.	Pos.	Heartworm and subcutaneous dirofilariosis
8	BSM	4.5	3	Neg.	Neg.	Neg.	Pos.	NE	Pos.	Pos.	Neg.	Pos.	Pos.	Occult heartworm and subcutaneous dirofilariosis
9	GS	3.5	2.5	Neg.	Neg.	Neg.	Neg.	NE	Neg.	Neg.	Neg.	Neg.	Neg.	Healthy dog
10	BSM	4	2	*D. repens*	Neg.	Neg.	Pos.	*D. repens*	Neg.	Pos.	Neg.	Pos.	Pos.	Occult heartworm and subcutaneous dirofilariosis
11	BSM	3	2	Neg.	Neg.	Neg.	Neg.	NE	Neg.	Neg.	Neg.	Neg.	Neg.	Healthy dog
12	GS	3	2	Neg.	Neg.	Neg.	Neg.	NE	Neg.	Neg.	Neg.	Neg.	Neg.	Healthy dog
13	GS	4	1.5	*D. repens*	Pos.	Pos.	Pos.	*D. repens*	Neg.	Pos.	Neg.	Pos.	Pos.	Occult heartworm and subcutaneous dirofilariosis
14	GS	2.5	1.5	Neg.	Neg.	Neg.	Neg.	NE	Neg.	Neg.	Neg.	Neg.	Neg.	Healthy dog
15	BSM	3.5	1	Neg.	Neg.	Neg.	Neg.	NE	Neg.	Neg.	Neg.	Neg.	Neg.	Healthy dog
16	BSM	2	0.5	Neg.	Neg.	Neg.	Neg.	NE	Neg.	Neg.	Neg.	Neg.	Neg.	Healthy dog
17	BSM	2	0.5	Neg.	Neg.	Neg.	Neg.	NE	Neg.	Neg.	Neg.	Neg.	Neg.	Healthy dog

* BSM: Belgian shepherd malinois, GS: German shepherd.

** NE: Not evaluated.

**Figure 1:**
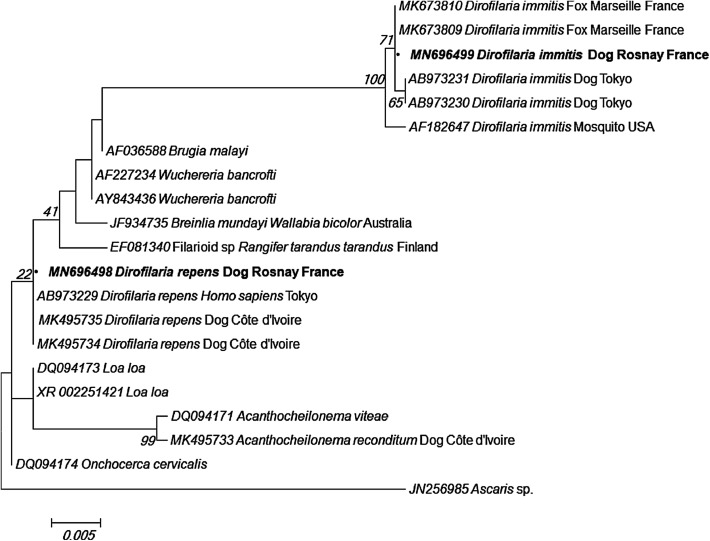
Molecular phylogenetic analysis of the 18S rRNA gene, using the maximum likelihood method based on the Kimura 3-substitution-type model.

Four partial sequences of the 18S rRNA gene have been successfully generated from samples identified as positive for a single DNA of filaria. Phylogenetic analysis revealed that dog No. 3 was infected with *D. immitis,* having 100% identity with *D. immitis* isolated from foxes in France (Marseille) (MK673809, MK673810) and 99.9% with that isolated from dogs in Japan (AB973231). Three *D. repens* sequences were 100% identical both to each other and to *D. repens* isolated from humans in Japan (Fig. 1).

**Figure 2:**
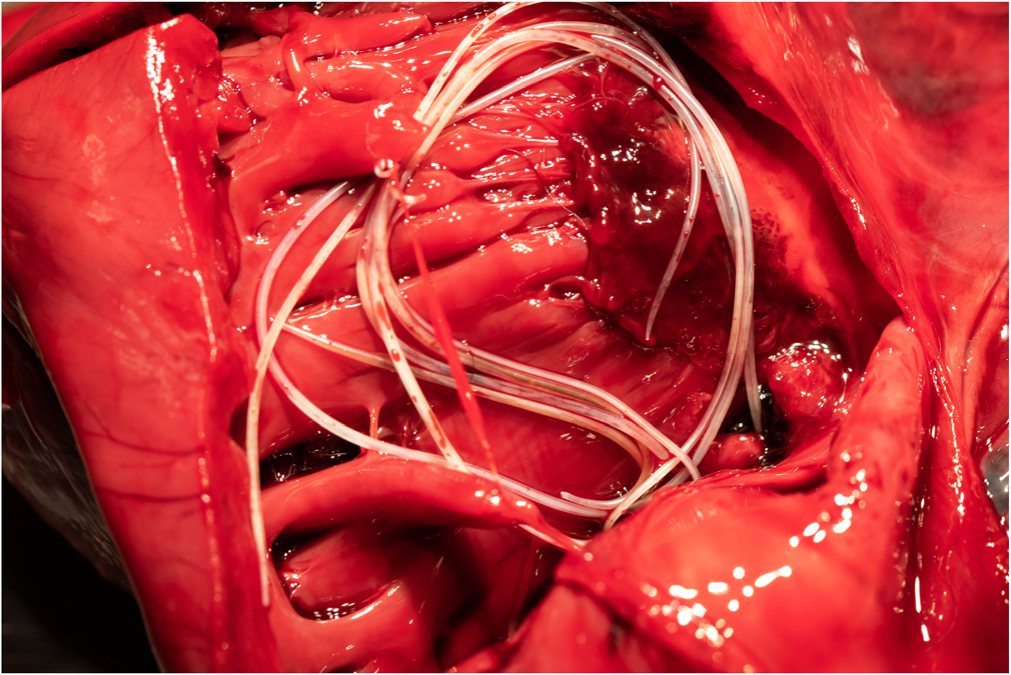
Heartworm (*Dirofilaria immitis*) in the right ventricle of dog No. 7.

Dog No. 7 died suddenly in November 2018 (1 month after blood sampling for our study) because of a stomach dilation-torsion. The necropsy performed on this dog revealed the presence in the right heart of four females and two males of *D. immitis* (Fig. 2). The heart was not dilated.

It is known that heartworm can occur as an occult infestation, resulting in the presence of at least one mature female (or male) of *D. immitis,* without circulating microfilariae. Occult infestations occur in several situations, including monosexual infestation by male or female worms only, infertility of female worms, low infestation levels and/or destruction of microfilariae due to the host’s immune response [35]. In our study, the proportion of occult infestation among dogs infected by *D. immitis* was 66.6% (4/6). The detection of occult infections is usually based on adult worm antigen testing [24]. However, in areas endemic for both *D. immitis* and *D. repens,* the reliability of these tests decreases, due to two main factors: (i) cross-reaction of rapid diagnostic tests between *D. immitis* and other parasites, including *D. repens*, and (ii) the immunosuppressive capacity of *D. repens* over *D. immitis* microfilariae, which hinders diagnosis based on the detection of blood microfilariae [14]. Adulticide treatment of *D. immitis* dirofilariasis, according to the protocol recommended by the American Heartworm Society, was effective against *D. repens* [28]. The approach combining the specific detection of molecular markers of *D. immitis* and *D. repens* showed 100% and 99.3% sensitivity and specificity, respectively. The reliability of the method is not reduced, even in case of occult dirofilariasis possibly accompanied by infestation by other filaroid species like *Acanthocheilonema reconditum*, leading to false positives following cross-reactions of rapid detection tests for *D. immitis* antigens [24]. This is coherent with the results obtained in our study, where occult heartworm infection associated with circulating microfilariae of *D. repens* was observed in 60% (3/5) of *Dirofilaria* spp. coinfected dogs, of which only 40% (2/5) were positive for *D. immitis* antigen.

Canine filarial infections have increased significantly in recent years [35]. This trend is the consequence of the increase in the range of vectors, as well as the introduction of infected dogs, reservoirs of parasites, into ecosystems favourable to the emergence of secondary indigenous outbreaks. The geographical distribution of heartworm disease and subcutaneous dirofilariosis in France is not precisely known. However, several studies reported that *D. repens* is more endemic and spreads more rapidly than *D. immitis* within northern and eastern Europe [2, 3, 14, 31, 36]. These differences in the epidemiology of the two parasite species could be explained by the fact that in areas where *D. repens* is widespread, the progression of *D. immitis* would be hindered, and *vice versa* [14]. Outbreaks of *D. immitis* have also been reported in western France, they concerned hunting dogs living in kennels near ponds [6, 7]. In our study, the military kennel is located in an infertile swampy area near seven ponds. The local ecosystem is highly favourable to the development of dirofilariosis vectors. *Aedes* (*Stegomyia*) *albopictus*, more commonly known as the “tiger mosquito”, has been introduced into metropolitan France from Italy and is now present throughout the south of the country, as well as in regions further north, including the Paris region and the department of Indre since 2017 [27]. This highly invasive and anthropophilic mosquito is known to be a competent vector of dirofilariosis parasites and to be implicated in the transmission of dirofilariosis in the studied area and most likely contributed to the infection of the military dogs considered in this study [36]. In southern Italy, the worrying increase in the proportion of mosquitoes infected by *D. immitis* among *A. albopictus* populations is associated with an increased risk of infestation of dogs with this parasite [15]. An integrated approach to control dirofilariosis vectors and to reduce infection sources and reservoirs for the parasite should be implemented in these areas. In the highly endemic area, the multi-modal prophylactic strategy, consisting in the administration of macrocyclic lactones and the application of repellents effective against mosquitoes, appears to be a tailored strategy.

A wide range of mammalian hosts, including humans and cats, can be infected by both *D. immitis* and *D. repens*, resulting from their low host specificity. Human infestation by *D. immitis* results in a pulmonary form (nodules), the most severe but less frequent form, which is found mainly in southern Europe (Italy, Spain, Greece, etc.) [13]. In France, most cases of human dirofilariasis caused by *D. immitis* are reported in endemic regions, such as Corsica and the Bouches-du-Rhône department [9, 32]. In humans, like in dogs, *D. repens* causes subcutaneous filariasis [1, 19].

We report here for the first time, an outbreak of canine dirofilariosis (*D. repens* and *D. immitis*) in the Rosnay military kennel, with a health risk for military personnel and military dogs in this area. In late 2018, after the detection of several infected dogs in the kennel, the following management measures were implemented: all dogs infected by *D. immitis* and cases of mixed infection (*N* = 6), except one that died before the treatment was administered (dog No. 7), received adulticide (melarsomine), combined with larvicide treatment (ivermectin), doxycycline and glucocorticosteroids (prednisone), according to the treatment protocol recommended by the American Heartworm Society [28]. During treatment, a restriction on physical activity was prescribed. This treatment eliminated *D. repens* and *D. immitis* larvae, as well as *Wolbachia* complex and the existing susceptible larvae. Moreover, all infected dogs were treated with insecticidal repellent effective against mosquitoes during the period of vector activity (from May to November in mainland France). The aim was to prevent secondary cases and outbreaks, in the presence of competent vectors in the area where the military kennel is located.

## Conclusions

The epidemiology of dirofilariosis infections is complex and has even been related to the concept of the episystem, represented by the multiple interactions between climate, environment, animals, humans and parasites [34]. As these are zoonoses, physicians and veterinarians must be informed of the risks of transmission in the regions, and especially the local biotopes [10]. We suggest that epidemiological investigations of vectors and canine dirofilariasis be implemented in areas at risk of exposure. Moreover, we suggest implementation of prevention against *D. immitis* and *D. repens* infection in dogs, using macrocyclic lactones [35], in combination with a repellent effective against mosquitoes (permethrin or deltamethrin-based products), as recommended for heartworm prevention in the affected and surrounding areas [28].

## References

[1] Benzaquen M, Brajon D, Delord M, Yin N, Bittar F, Toga I, Berbis P, Parola P. 2015 Cutaneous and pulmonary dirofilariasis due to *Dirofilaria repens*. British Journal of Dermatology, 173, 788–791. 25918821 10.1111/bjd.13859

[2] Capelli G, Genchi C, Baneth G, Bourdeau P, Brianti E, Cardoso L, Danesi P, Fuehrer HP, Giannelli A, Ionicǎ AM, Maia C, Modrý D, Montarsi F, Krücken J, Papadopoulos E, Petrić D, Pfeffer M, Savić S, Otranto D, Poppert S, Silaghi C. 2018 Recent advances on *Dirofilaria repens* in dogs and humans in Europe. Parasites and Vectors, 11(1), 663. 30567586 10.1186/s13071-018-3205-x PMC6299983

[3] Chauve CM. 1997 Importance in France of the infestation by *Dirofilaria* (*Nochtiella*) *repens* in dogs. Parassitologia, 39(4), 393–395. 9802099

[4] Davoust B, Ducos de Lahitte J. 1986 Utilisation de l’ivermectine dans la prophylaxie de la dirofilariose canine : résultats obtenus après deux ans d’essais chez le chien militaire en zone enzootie. Bulletin de la Société Française de Parasitologie, 4(2), 235–239.

[5] Davoust B, Ducos de Lahitte J. 1989 Évolution de l’enzootie de dirofilariose dans les chenils militaires du Sud-Est de la France. Revue de Médecine Vétérinaire, 140(1), 15–19.

[6] Doby JM, Couatarmanach A, Aznar C. 1986 Filarioses canines par *Dirofilaria immitis* (Leidy, 1856) et *D. repens* (Railliet et Henry, 1911), dans l’ouest de la France. Bulletin de la Société Française de Parasitologie, 4(2), 229–233.

[7] Doby JM, Guiguen C. 1986 Présence de *Dirofilaria immitis* en Bretagne. Bulletin de la Société Française de Parasitologie, 4, 51–54.

[8] Ducos de Lahitte J, Davoust B, Dorchies P. 1984 Filariose canine à *Dirofilaria immitis.* Enquête sur la fréquence et la répartition en zone méditerranéenne. Bulletin de la Société Française de Parasitologie, 3, 105–108.

[9] Foissac M, Million M, Mary C, Dales JP, Souraud JB, Piarroux R, Parola P. 2013 Subcutaneous infection with *Dirofilaria immitis* nematode in human, France. Emerging Infectious Diseases, 19(1), 171–172. 23260094 10.3201/eid1901.120281 PMC3557977

[10] Genchi C, Bowman D, Drake J. 2014 Canine heartworm disease (*Dirofilaria immitis*) in Western Europe: survey of veterinary awareness and perceptions. Parasites and Vectors, 7, 206. 24779376 10.1186/1756-3305-7-206 PMC4013803

[11] Genchi C, Kramer L. 2017 Subcutaneous dirofilariosis (*Dirofilaria repens*): an infection spreading throughout the old world. Parasites and Vectors, 10(Suppl 2), 517. 29143643 10.1186/s13071-017-2434-8 PMC5688444

[12] Genchi C, Kramer LH, Rivasi F. 2011 Dirofilarial infections in Europe. Vector-Borne and Zoonotic Diseases, 11(10), 1307–1317. 21417922 10.1089/vbz.2010.0247

[13] Genchi C, Mortarino M, Rinaldi L, Cringoli G, Traldi G, Genchi M. 2011 Changing climate and changing vector-borne disease distribution: the example of Dirofilaria in Europe. Veterinary Parasitology, 176, 295–299. 21300439 10.1016/j.vetpar.2011.01.012

[14] Genchi C, Rinaldi L, Cascone C, Mortarino M, Cringoli G. 2005 Is heartworm disease really spreading in Europe? Veterinary Parasitology, 133(2–3), 137–148. 15885913 10.1016/j.vetpar.2005.04.009

[15] Giangaspero A, Marangi M, Latrofa MS, Martinelli D, Traversa D, Otranto D, Genchi C. 2013 Evidences of increasing risk of dirofilarioses in southern Italy. Parasitology Research, 112(3), 1357–1361. 23224639 10.1007/s00436-012-3206-1

[16] Hall TA. 1999 BIOEDIT: a user-friendly biological sequence alignment editor and analysis program for Windows 95/98/NT. Nucleic Acids Symposium Series, 95–98.10780396

[17] Henry LG, Brunson KJ, Walden HS, Wenzlow N, Beachboard SE, L Barr K, Long MT. 2018 Comparison of six commercial antigen kits for detection of *Dirofilaria immitis* infections in canines with necropsy-confirmed heartworm status. Veterinary Parasitology, 254, 178–182. 29657005 10.1016/j.vetpar.2018.02.037

[18] Joyeux C, Cabassu J. 1935 Étude sur la filariose des chiens de Camargue. Bulletin de la Société de Pathologie Exotique, 28, 187–193.

[19] Kartashev V, Batashova I, Kartashov S, Ermakov A, Mironova A, Kuleshova Y, Ilyasov B, Kolodiy I, Klyuchnikov A, Ryabikina E, Babicheva M, Levchenko Y, Pavlova R, Pantchev N, Morchón R, Simón F. 2011 Canine and human dirofilariosis in the Rostov Region (Southern Russia). Veterinary Medicine International, 2014, 6857 13. 10.4061/2011/685713 PMC3022198 21253482

[20] Kimura M. 1981 Estimation of evolutionary distances between homologous nucleotide sequences (molecular evolution/comparison of base sequences/base substitution rate/neutral mutation-random drift hypothesis). Genetics, 78, 454–458. 10.1073/pnas.78.1.454 PMC319072 6165991

[21] Knott J. 1939 A method for making microfilarial surveys on day blood. Transactions of the Royal Society of Tropical Medicine and Hygiene, 33, 191–196.

[22] Laidoudi Y, Davoust B, Varloud M, Niang EHA, Fenollar F, Mediannikov O. 2019 Development of a multiplexed qPCRs-based approach for the diagnosis of *Dirofilaria immitis, D. repens, Acanthocheilonema reconditum* and the others filariosis. bioRxiv 842575; 10.1101/842575. PMC7309989 32571427

[23] Leidy J. 1856 A synopsis of entozoan and some of the other ecto-congeners observed by the author. Proceedings of Academy National of Sciences of Philadelphia, 8, 42–58.

[24] Little S, Saleh M, Wohltjen M, Nagamori Y. 2018 Prime detection of *Dirofilaria immitis*: understanding the influence of blocked antigen on heartworm test performance. Parasites and Vectors, 11, 1–10. 29554955 10.1186/s13071-018-2736-5 PMC5859648

[25] Magnis J, Lorentz S, Guardone L, Grimm F, Magi M, Naucke TJ, Deplazes P. 2013 Morphometric analyses of canine blood microfilariae isolated by the Knott’s test enables *Dirofilaria immitis* and *D. repens* species-specific and *Acanthocheilonema* (syn. *Dipetalonema*) genus-specific diagnosis. Parasites and Vectors, 6, 48. 23442771 10.1186/1756-3305-6-48 PMC3598535

[26] Mccall JW, Genchi C, Kramer LH, Guerrero J, Venco L. 2008 Heartworm disease in animals and humans. Advances in Parasitology, 66, 193–285. 18486691 10.1016/S0065-308X(08)00204-2

[27] Ministère des Solidarités et de la Santé (France). 2019 Cartes de présence du moustique tigre (*Aedes albopictus*) en France métropolitaine. Web site (updated 28.05.19). https://solidarites-sante.gouv.fr/sante-et-environnement/risques-microbiologiques-physiques-et-chimiques/especes-nuisibles-et-parasites/article/cartes-de-presence-du-moustique-tigre-aedes-albopictus-en-france-metropolitaine.

[28] Nelson CT, McCall JW, Jones S, Moorhead A. 2018 Current canine guidelines for the prevention, diagnosis, and management of Heartworm (*Dirofilaria immitis*) infection in dogs. Wilmington, DE: American Heartworm Society p. 1–35.

[29] Otranto D, Dantas-Torres F, Brianti E, Traversa D, Petrić D, Genchi C, Capelli G. 2013 Vector-borne helminths of dogs and humans in Europe. Parasites and Vectors, 6, 16. 23324440 10.1186/1756-3305-6-16 PMC3564894

[30] Panthot JB. 1679 Extrait d’une lettre écrite de Lyon à l’auteur du Journal par Monsieur Panthot D. en Méd. & Professeur aggrégé au Collège de Lyon, contenant deux observations remarquables. Journal des Sçavans, 238.

[31] Pantchev N, Schaper R, Limousin S, Norden N, Weise M, Lorentzen L. 2009 Occurrence of *Dirofilaria immitis* and tick-borne infections caused by *Anaplasma phagocytophilum, Borrelia burgdorferi sensu lato* and *Ehrlichia canis* in domestic dogs in France: results of a countrywide serologic survey. Parasitology Research, 105(Suppl 1), S101–S114. 19575231 10.1007/s00436-009-1501-2

[32] Raccurt CP. 1999 Les dirofilarioses, des zoonoses émergentes et sous-estimées en France. Médecine Tropicale, 59(4), 389–400. 10816755

[33] Railliet A, Henry A. 1911 Sur une filaire péritonéale des porcins. Bulletin de la Société de Pathologie Exotique, 4, 386–389.

[34] Simón F, González-Miguel J, Diosdado A, Gómez PJ, Morchón R, Kartashev V. 2017 The complexity of zoonotic filariasis episystem and its consequences: a multidisciplinary view. BioMed Research International, 2017, 6436130. 28642878 10.1155/2017/6436130 PMC5469992

[35] Simón F, Siles-Lucas M, Morchón R, González-Miguel J, Mellado I, Carretón E, Montoya-Alonso JA. 2012 Human and animal dirofilariasis: the emergence of a zoonotic mosaic. Clinical Microbiology Reviews, 25(3), 507–544. 22763636 10.1128/CMR.00012-12 PMC3416488

[36] Tahir D, Bittar F, Barré-Cardi H, Sow D, Dahmani M, Mediannikov O, Raoult D, Davoust B, Parola P. 2017 Molecular survey of *Dirofilaria immitis* and *Dirofilaria repens* by new real-time TaqMan^®^PCR assay in dogs and mosquitoes (Diptera: Culicidae) in Corsica (France). Veterinary Parasitology, 235, 1–7. 28215858 10.1016/j.vetpar.2017.01.002

[37] Tamura K, Stecher G, Peterson D, Filipski A, Kumar S. 2013 MEGA6: molecular evolutionary genetics analysis version 6.0. Molecular Biology and Evolution, 30(12), 2725–2729. 24132122 10.1093/molbev/mst197 PMC3840312

[38] Tarello W. 2011 Clinical aspects of dermatitis associated with Dirofilaria repens in pets: a review of 100 canine and 31 feline cases (1990–2010) and a report of a new clinic case imported from Italy to Dubai. Journal of Parasitology Research, 2011, 578385. 22203888 10.1155/2011/578385 PMC3238394

